# Supplementary material for: 120 GOPS Photonic tensor core in thin-film lithium niobate for inference and in situ training
Source: Nat Commun. 2024 Oct 21;15:9081. doi: 10.1038/s41467-024-53261-x (PMC11493977; doi:10.1038/s41467-024-53261-x)
Supplement: Supplementary file 1 — Supplementary Information [file 41467_2024_53261_MOESM1_ESM.pdf]

Supporting information for "120 GOPS Photonic Tensor Core in Thin-film Lithium Niobate for Inference and in-situ Training"

Zhongjin Lin[1,2], Bhavin J. Shastri[3], Shangxuan Yu[1], Jingxiang Song[1], Yuntao Zhu[2], Arman Safarnejadian[4], Wangning Cai[1], Yanmei Lin[2], Wei Ke[2], Mustafa Hammood[1], Tianye Wang[1], Mengyue Xu[2], Zibo Zheng[4], Mohammed Al-Qadasi[1], Omid Esmaeeli[1], Mohamed Rahim[5], Grzegorz Pakulski[5], Jens Schmid[5], Pedro Barrios[5], Weihong Jiang[5], Hugh Morison[3], Matthew Mitchell[1], Xun Guan[6], Nicolas A. F. Jaeger[1], Leslie A. Rusch[4], Sudip Shekhar[1], Wei Shi[4], Siyuan Yu[2], Xinlun Cai[2*] and Lukas Chrostowski[1*]

[1]Department of Electrical and Computer Engineering, The University of British Columbia, Vancouver, V6T 1Z4, British Columbia, Canada.
[2]State Key Laboratory of Optoelectronic Materials and Technologies, School of Electronics and Information Technology, Sun Yat-sen University, Guangzhou, 510275, Guangdong, China.
[3]Department of Physics, Engineering Physics and Astronomy, Queen's University, Kingston, K7L 3N6, Ontario, Canada.
[4]Department of Electrical and Computer Engineering, Université Laval, Québec City, G1V 0A6, Québec, Canada.
[5]Advanced Electronics and Photonics Research Centre, National Research Council, Ottawa, K1A 0R6, Ontario, Canada.
[6] Tsinghua-Berkeley Shenzhen Institute, Tsinghua University, Shenzhen, 581055, China.

*Corresponding author(s). E-mail(s): caixlun5@mail.sysu.edu.cn; lukasc@ece.ubc.ca;

1 Design of device

Figure S1a presents a schematic of the proposed device, including laser, photodetectors, fiber array, and thin-film lithium niobate (TFLN) chip. The TFLN chip includes 10 optical input/output (I/O) ports: 1 port is used for connecting with the laser; 7 ports are used for connecting with the fiber array for measuring the electro-optics (EO) bandwidth of modulators, and calibrating bias voltages and delay time; 2 ports are used for coupling light form waveguide onto the photodetectors through grating couplers. The wavelength emitted from the laser is 1310 nm. The layout of the TFLN chip used for the fabrication is shown in Fig. S1b.

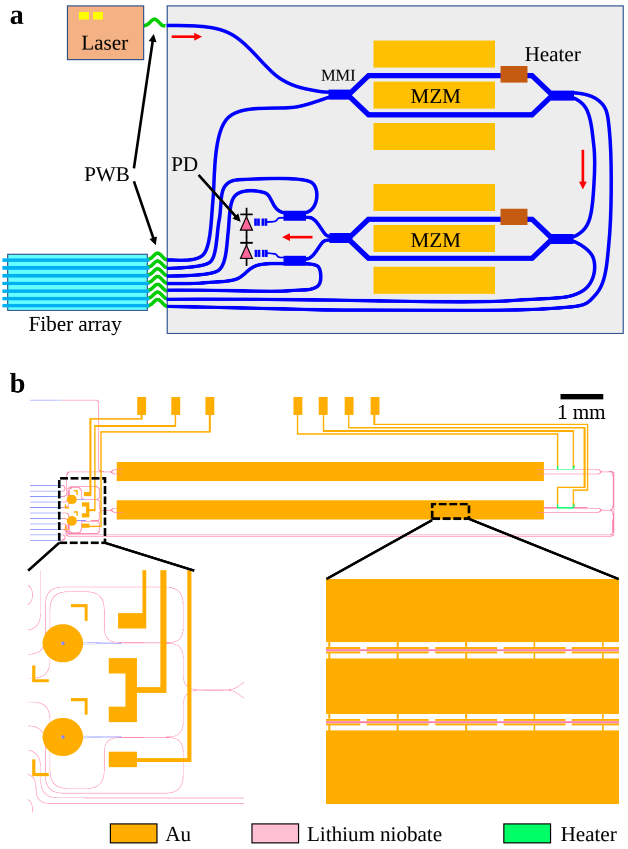

Fig. S1 The structure of the proposed device. a Schematic of the proposed device including laser, photodetectors, and thin-film lithium niobate (TFLN) chip. PWB: photonic wire bonding; MMI: 3-dB multimode interference; MZM: Mach-Zehnnder modulator. **b** Schematic of the TFLN chip.

2 Performance of hybrid integrated laser

Figure S2 shows the transmission loss between a thin-film lithium niobate edge coupler and a single-mode fiber through a photonic wire bond (PWB), indicating a minimum insertion loss of 1.6 dB and a 1 dB bandwidth larger than 110 nm.

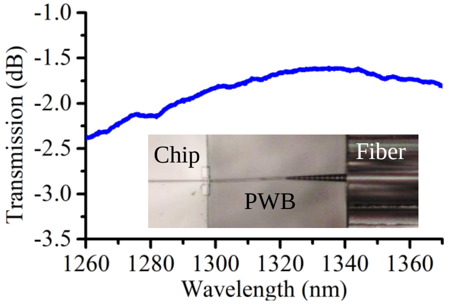

Fig. S2 The transmission loss between a thin-film lithium niobate edge coupler and a single-mode fiber through a photonic wire bond (PWB). Inset: a micrograph of PWB chip-to-fiber interconnect.

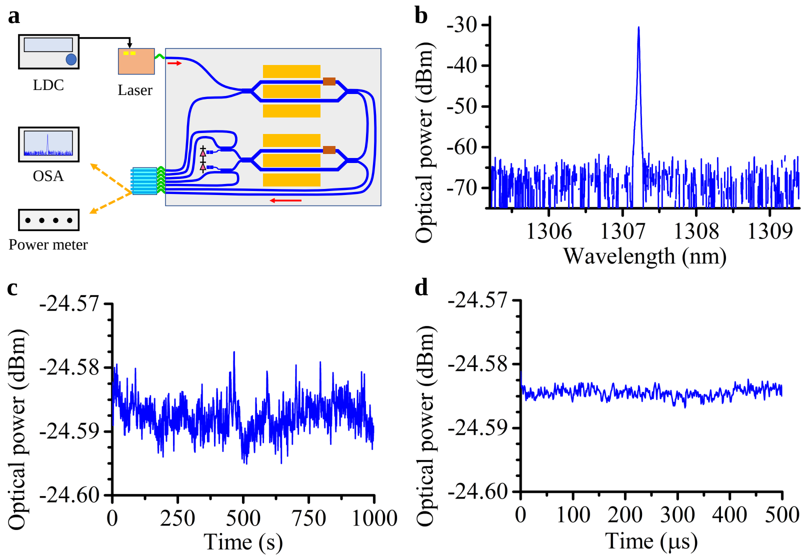

Fig. S3 Performance of the hybrid integrated laser. a Schematic of the experimental setup for characterizing the performance of the hybrid integrated laser. LDC: laser diode controller; OSA: optical spectrum analyzer. **b** The measured laser spectrum when setting the current applied to the laser at 30 mA. The side mode suppression ratio of the laser is larger than 35 dB. **c** and **d** are the output optical powers of laser varying with time when the sampling rates of a power meter are set at 1 sample per second and 10^6 samples per second, respectively. The output power variation is less than 0.02 dB within 1000 seconds.

Next, we characterized the performance of our hybrid integrated laser. Figure S3 presents a schematic of the experimental setup. Our laser was driven by a laser diode controller (LDC, LDC501 SRS), and measured by an optical spectrum analyzer (OSA, AQ6317B, ANDO) and a power meter (Agilent N7744A). Here, we did not apply any electronic signal to our TFLN chip to avoid the influence of additional noise on laser measurement, and thus optical output power received by OSA and power meter is low. Figure S3 shows a laser spectrum when the drove current was set at 30 mA. Note that we also set the drove current at 30 mA when we performed dot product, classification, and clustering experiments. The side mode suppression ratio of our laser is larger than 35 dB. Figures S3c and S3d show the variation of laser output power with time. The power stability of our laser is less than 0.02 dB within 1000 seconds which is enough for us to perform dot product, classification, and clustering experiments.

3 Design of electrical controlling circuit for photoreceiver

Figure S4a presents a schematic of an electrical controlling circuit for our photoreceiver. Two photodetectors, in a balanced detection scheme, connect with an integrator and then connect with an analog-to-digital converter (ADC) through a voltage follower. The ADC and integrator are controlled by a microcontroller (MC). By controlling the switches (marked as S1 and S2) of the integrator, the working status of the integrator can be changed:

(1) Closing S1 and opening S2, photogenerated electrons from PD1 and PD2 can be accumulated in the capacitance of integrator;

(2) Simultaneously opening S1 and S2, the output voltage of the integrator, *i.e.*, the number of accumulated electrons, can be read by ADC and MC;

(3) Simultaneously closing S1 and S2, the accumulated electrons in the capacitance of the integrator can be reset.

In a balanced detection scheme, when the optical power received by PD1 is lower than that received by PD2, the output voltage variation of the integrator is positive and, when it is higher than that received by PD2, the output voltage variation of the integrator is negative.

Figure S4b shows a photo of the fabricated electrical controlling circuit board. The integrator and voltage follower are covered with a metal shield to avoid the electromagnetic interference and decrease the noise. Our electrical controlling circuit board is connected with computer through USB cable for power supply and communication. Using USB bus converter chip (CH343), the USB can be converted to serial port, and thus our electrical controlling circuit can be controlled by a Python command.

Figure S4c shows a schematic of part of the electrical controlling circuit. The circuit includes an integrator (IVC102U), a voltage follower (ADA4522ARMZ), a differential driver (AD8131), an ADC (AD7767), and a microcontroller (STC8A8K64D4_LQFP44).

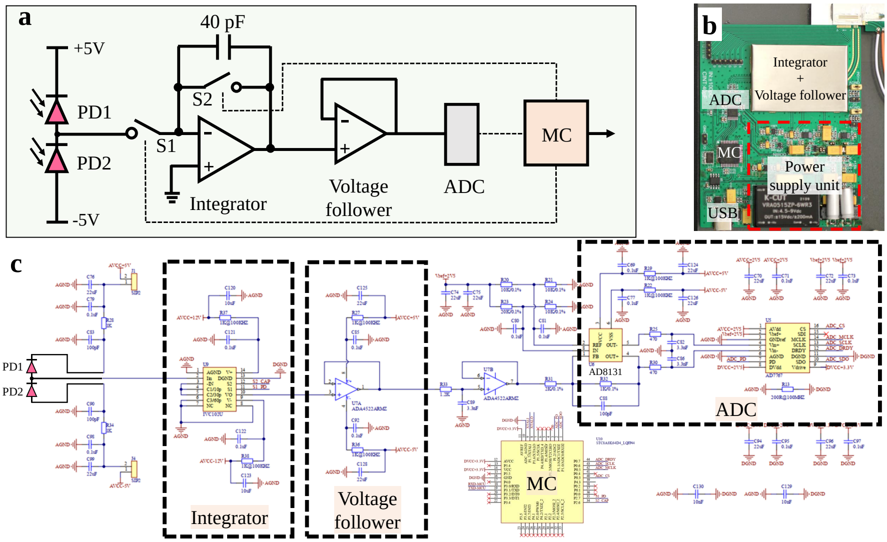

Fig. S4 Design of electrical controlling circuit. a a schematic of an electrical controlling circuit for the proposed integrator. **b** A photo of the fabricated electrical controlling circuit board. ADC: analog-to-digital converter; MC: microcontroller. **c** Layout of the electrical controlling circuit without the power supply unit part.

4 Experiment

Figure S5a presents a schematic of the experimental setup for characterizing the performance of our device. We used high-speed digital-to-analog converters (DACs, Keysight, M8194A) to drive our modulators. Here, although we used RF amplifiers (Inphi, IN3214SZ-S02G) to apply gain to the output power of the high-speed DACs, in the future, our device can be directly driven by the high-speed DACs using TFLN modulators which have a lower half-wave voltage [1]. Two tunable phase shifters (SPECTRUM Microwave, MODEL OPS-0002) were used to tune the delay time between two TFLN modulators. A computer was used to connect to the high-speed DAC and the electrical controlling circuit board of the integrator. In the future, the high-speed DACs and the integrator can be monolithically integrated into a single chip to achieve a compact size.

We use Python, running on the CPU, to control all devices. As illustrated in Fig. S5b of the revised supplementary materials, when a matrix-vector multiplication operation is required, the CPU sends a "Start to Integration" command to the charge integrator. However, at this point, no charge is being accumulated due to the balancing scheme. Subsequently, the CPU sends a "Start to Encode" command to the DACs. Once all data is encoded, the CPU sends an "End to Integration" command to the charge integrator. The integrated circuit (IC) then returns the voltage of the charge integrator to the CPU sequentially, enabling the retrieval of the matrix-vector multiplication result.

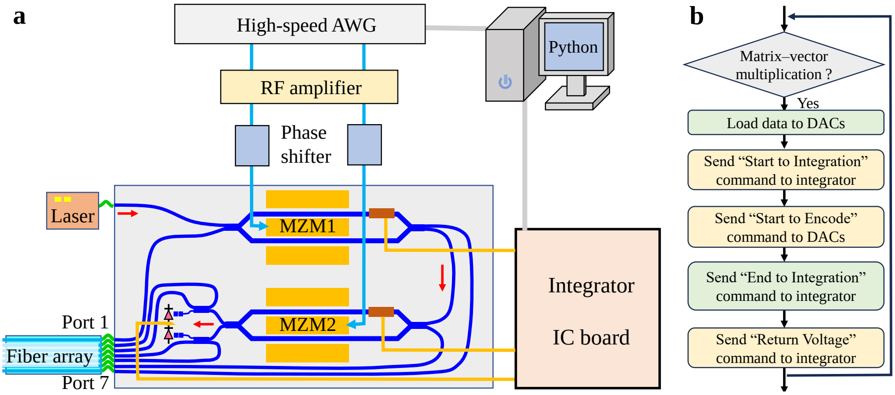

Fig. S5 Experimental setup for characterizing the performance of our device. **a** A schematic of the experimental setup used for dot product demonstration, classification, and clustering. DAC: digital to analog converter. **b** Algorithm process flow chart.

4.1 System calibration

7 of the optical I/O ports of our TFLN chip connected with a fibre array are used for measuring the EO bandwidth of modulators, and calibrating bias voltages and delay time between two modulators. As shown in Fig. S5a, we label the 7 ports from up to down as port 1, port 2,..., and port 7, respectively.

Bias voltages calibration and EO bandwidth measurement. Connecting a high-power laser (Santec TSL550) with port 1 (or port 6) and connecting a high-speed photodetector (Finisar, XPDV3120R) with port 7 (or port 4), we can characterize the EO response of the fabricated modulator at a telecom wavelength of 1310 nm using a vector network analyzer (Agilent N5227A). The bias voltages also can be calibrated through the same connections but using a low-speed photodetector instead of a high-speed one.

Calibration of delay time between two modulators. Connecting a high-power laser (Santec TSL550) with port 1 and connecting an oscilloscope with port 4, the two modulators were simultaneously driven by CMOS DAC. Tuning the phase shifter based on the data displayed on the oscilloscope, we can calibrate the delay time between two modulators to guarantee that the input and weight sequences are aligned and thus each weight vector element can be correctly multiplied by the corresponding element of the input vector.

4.2 Dot product operation

The bias points of two modulators were chosen to be set at the orthogonal points. The steps of using our device to perform dot product:

(1) Simultaneously closing S1 and S2;

(2) Setting the integration time of the integrator, and then closing S1 and opening S2;

(3) Sending two data sequences with a length of n to CMOS DACs, and driving two modulators with a baud rate of f_s;

(4) Opening S1 and reading the output voltage of the integrator;
(5) Repeat step (1) for a new sequence.

4.3 Experiment of classification task

The test accuracy as a function of the effective resolution used in the matrix-vector multiplication operations is shown in Fig. S6a. These simulation results demonstrate that the training process is robust to noise and low-precision computations. Therefore, although our processor only has a resolution of 6.04 bits, it is sufficient for training. The validation accuracy during training, using both our processor and a CPU (for comparison), is depicted in Fig. S6b. This demonstrates that the "hardware-in-the-loop" training approach can achieve a convergence speed comparable to that of training solely on the CPU.

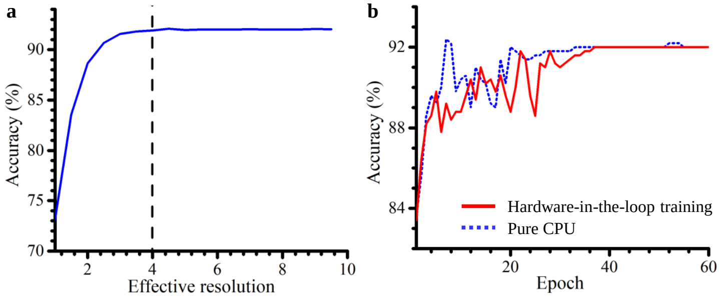

Fig. S6 **a** Validation accuracy on the MNIST dataset as a function of the effective resolution. **b** The convergence over epoch for both "hardware-in-the-loop" training and pure central processing unit (CPU).

4.4 Experimental setup for clustering task

Here, we show how to perform the dot product operation between two vectors with ranges from -1 to 1. As discussed earlier, it is straightforward to achieve a range of -1 to 1 for the vector modulated by the second modulator. However, the vector modulated by the first modulator can only achieve a range of 0 to 1. To address this, we need to inject light with a wavelength of λ_2 into the second modulator through the optical port 6, as shown in Fig. S7. We defined the voltages applied to the two modulators are V_1 and V_2, respectively. The electrical field intensity of light injected into the first modulator is set at E_{0,λ_1} with a wavelength λ_1, and thus the output electrical field intensity of the first modulator can be given by,

$$E_{1,\lambda_1} = E_{0,\lambda_1} \cos(\frac{V_1 \pi}{2V_\pi(\lambda_1)} + \frac{\theta_1}{2}), \tag{S1}$$

, where $V_\pi(\lambda_1)$ is half-wave voltage of the modulator at wavelength λ_1. θ_1 is the bias phase difference of the first modulator. The output electrical field

intensities of the second modulator, E_3 and E_4, can be given by,

$$E_3 = jE_{1,\lambda_1}\sin(\frac{V_2\pi}{2V_\pi(\lambda_1)} + \frac{\theta_2}{2}) + E_{2,\lambda_2}\cos(\frac{V_2\pi}{2V_\pi(\lambda_2)} + \frac{\theta_2}{2}), \tag{S2}$$

$$E_4 = E_{1,\lambda_1}\cos(\frac{V_2\pi}{2V_\pi(\lambda_1)} + \frac{\theta_2}{2}) + jE_{2,\lambda_2}\sin(\frac{V_2\pi}{2V_\pi(\lambda_2)} + \frac{\theta_2}{2}), \tag{S3}$$

, where $V_\pi(\lambda_2)$ is half-wave voltage of the modulator at wavelength λ_2. θ_2 is the bias phase difference of the first modulator. Because λ_1 and λ_2 are close, $V_\pi(\lambda_2) = V_\pi(\lambda_1) = V_{\pi,0}$ [2]. We can obtain that,

$$\mid E_3 \mid^2 - \mid E_4 \mid^2 = (- \mid E_{1,\lambda_1} \mid^2 + \mid E_{2,\lambda_2} \mid^2)\cos(\frac{V_2\pi}{V_{\pi,0}} + \theta_2). \tag{S4}$$

We set $\mid E_{2,\lambda_2} \mid^2 = \frac{|E_{0,\lambda_1}|^2)}{2}$. Combining Eq. S1 and Eq. S4, we can obtained that,

$$\mid E_3 \mid^2 - \mid E_4 \mid^2 = -\frac{\mid E_{0,\lambda_1} \mid^2}{2}\cos(\frac{V_1\pi}{V_\pi(\lambda_1)} + \theta_1)\cos(\frac{V_2\pi}{V_{\pi,0}} + \theta_2). \tag{S5}$$

If the bias points of two modulators are set at the orthogonal points, we can obtain that,

$$\mid E_3 \mid^2 - \mid E_4 \mid^2 = -\frac{\mid E_{0,\lambda_1} \mid^2}{2}\sin(\frac{V_1\pi}{V_{\pi,0}})\sin(\frac{V_2\pi}{V_{\pi,0}}). \tag{S6}$$

According to Eq. S6, this method allows us to perform the dot product operation between two vectors whose ranges are from -1 to 1, as opposed to one vector ranging from 0 to 1 and the other vector ranging from -1 to 1.

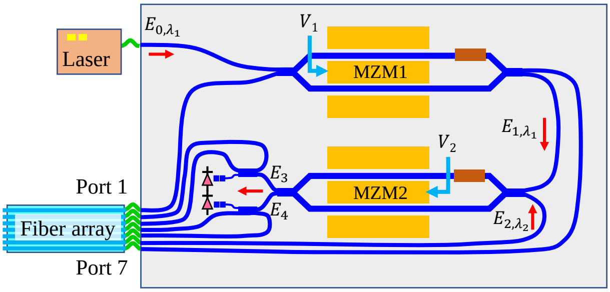

Fig. S7 Experimental setup for performing clustering task.

Table S1 Comparison of different modulator technologies employed in our proposed photonic processor which includes two modulators.

Technology	SiGe EAM [3]	InP MZM [4]	Si MZM [5]	Si MRM [6]	TFLN MZM [7]
Modulation speed (Gbaud/s)[a]	50	100	56	32.5	260
Length (mm)	0.61	4	4	0.02	10
Insertion loss (dB/cm)[b]	57	1.5	3	3	0.2
Compute density (GOPS/mm^2)	810	250	140	16250	260
Modulation loss?	N/A	Yes	Yes	Yes	No
Two negative number multiplication?	No	No	No	No	Yes
TDM&WDM? [c]	No	No	No	No	Yes

a. Modulation speed has been demonstrated;
b. Waveguide propagation loss;
c. Whether this technology can be used to build the architecture shown in Fig. 6 of the main manuscript.
N/A indicates no available data.

Table S2 Energy of our proposed photonic processor which includes two TFLN modulators.

Components	Energy budget	Energy efficiency (32.5 Gbaud)	Energy efficiency (60 Gbaud)
Laser driver [8]	15 mW	230 fJ/OP	125 fJ/OP
DAC (7nm FinFET) [9]	35 fJ/conv-step	35 fJ/OP	29 fJ/OP
Bias controller	17 mW	523 fJ/OP	56 fJ/OP [a]
Integrator [10]	44 μW	0.67 fJ/OP	0.37 fJ/OP
ADC (1 GHz) [10]	2.55 mW	39 fJ/OP	2.17 fJ/OP
Total	N/A	828 fJ/OP	213 fJ/OP

a. Heating efficiency can be improved up to 3 times higher than the presented structure [11].
N/A indicates no available data

5 Compared with other technology

Table S1 compares various modulator technologies—SiGe electro-absorption modulator (EAM), InP Mach-Zehnder modulator (MZM), Si MZM, Si microring modulator (MRM)—for our proposed processor architecture. Specifically, Table S1 compares factors like the compute density and insertion loss, among others. Notably, the photonic processor based on TFLN MZM stands out as

Table S3 Energy of our proposed photonic processor which includes $64 + 64$ TFLN modulators with a computation speed of 491 TOPS.

Components	Energy budget	Number	Energy efficiency (60 Gbaud)
Pump laser	1 W	1	2.04 fJ/OP
DAC (7nm FinFET) [9]	35 fJ/conv-step	$64 + 64$	0.59 fJ/OP
Bias controller	5.6 mW	$64 + 64$	1.46 fJ/OP [a]
Integrator [10]	44 μW	64×64	0.37 fJ/OP
CMOS comparator [12]	244.19 μW	64×64	2.04 fJ/OP
CMOS switch [13]	122 nW	64×64	0.001 fJ/OP
Total	N/A	N/A	6.5 fJ/OP

a. Heating efficiency can be improved up to 3 times higher than the presented structure [11].
N/A indicates no available data.

Table S4 Comparison of various optical computing architectures, both on-chip and in free space.

Sources	Energy efficiency[a]	Total neuron[a]	Tunable neuron	Network scale
This work[b]	153 TOPS/W	802,816	802,816	51 million
Feldmann et al. [14]	0.50 TOPS/W	64	64	29,186
Ashtiani et al. [15]	2.9 TOPS/W	67	67	67
Shen et al. [16]	N/A	213	213	1065
Zhou et al. [17]	0.71 TOPS/W	490,000	490,000	1.7 million
Xu et al. [18]	N/A	N/A	867	867
NVIDIA H100 PCIe [19]	0.71 TOPS/W	N/A	N/A	N/A

a. By theoretical calculations.
b. Based on our proposed photonic processor which includes $64 + 64$ TFLN modulators.
N/A indicates no available data.

the only one capable of multiplying with two negative numbers and is fully compatible with hybrid TDM and WDM architectures.

6 Energy consumption

In theory, for a computation speed of 120 GOPS, our device can achieve an energy efficiency of 213 fJ/OP (see Table S2), including the laser driver, DAC, bias voltage controller, op-amp integrator, and ADC. Moreover, as shown in Table 3, the proposed scalable photonic tensor core, which includes 64 + 64

TFLN modulators, can achieve an energy efficiency of 6.5 fJ/OP with a computational speed of 491 TOPS (*i.e.*, 153 TOPS/W). We also compare our proposed solution with some other optical processors in Table S4.

References

[1] Xu, S., Wang, J., Shu, H., Zhang, Z., Yi, S., Bai, B., Wang, X., Liu, J., Zou, W.: Optical coherent dot-product chip for sophisticated deep learning regression. Light: Science & Applications **10**(1), 221 (2021)

[2] Ke, W., Lin, Y., He, M., Xu, M., Zhang, J., Lin, Z., Yu, S., Cai, X.: Digitally tunable optical delay line based on thin-film lithium niobate featuring high switching speed and low optical loss. Photonics Research **10**(11), 2575–2583 (2022)

[3] Giamougiannis, G., Tsakyridis, A., Moralis-Pegios, M., Mourgias-Alexandris, G., Totovic, A.R., Dabos, G., Kirtas, M., Passalis, N., Tefas, A., Kalavrouziotis, D., *et al.*: Neuromorphic silicon photonics with 50 GHz tiled matrix multiplication for deep-learning applications. Advanced Photonics **5**(1), 016004–016004 (2023)

[4] Lange, S., Wolf, S., Lutz, J., Altenhain, L., Schmid, R., Kaiser, R., Schell, M., Koos, C., Randel, S.: 100 GBd intensity modulation and direct detection with an InP-based monolithic DFB laser Mach–Zehnder modulator. Journal of Lightwave Technology **36**(1), 97–102 (2018)

[5] Li, M., Wang, L., Li, X., Xiao, X., Yu, S.: Silicon intensity Mach–Zehnder modulator for single lane 100 Gb/s applications. Photonics Research **6**(2), 109–116 (2018)

[6] Huang, C., Fujisawa, S., de Lima, T.F., Tait, A.N., Blow, E.C., Tian, Y., Bilodeau, S., Jha, A., Yaman, F., Peng, H.-T., *et al.*: A silicon photonic–electronic neural network for fibre nonlinearity compensation. Nature Electronics **4**(11), 837–844 (2021)

[7] Mardoyan, H., Almonacil, S., Jorge, F., Pittalà, F., Xu, M., Krueger, B., Blache, F., Duval, B., Chen, L., Yan, Y., *et al.*: First 260-GBd single-carrier coherent transmission over 100 km distance based on novel arbitrary waveform generator and thin-film lithium niobate I/Q modulator. In: European Conference and Exhibition on Optical Communication, pp. 3–2 (2022). Optica Publishing Group

[8] Sackinger, E., Ota, Y., Gabara, T.J., Fischer, W.C.: A 15-mw, 155-Mb/s CMOS burst-mode laser driver with automatic power control and end-of-life detection. IEEE Journal of Solid-State Circuits **35**(2), 269–275 (2000)

[9] Nguyen, R., Castrillon, A., Fan, A., Mellati, A., Reyes, B.T., Abidin, C.,

Olsen, E., Ahmad, F., Hatcher, G., Chana, J., *et al.*: A highly reconfigurable 40-97GS/s DAC and ADC with 40 GHz AFE bandwidth and sub-35fJ/conv-step for 400 Gb/s coherent optical applications in 7 nm FinFET. In: 2021 IEEE International Solid-State Circuits Conference (ISSCC), vol. 64, pp. 136–138 (2021). IEEE

[10] Yang, E., Lehmann, T.: High gain operational amplifiers in 22 nm CMOS. In: 2019 IEEE International Symposium on Circuits and Systems (ISCAS), pp. 1–5 (2019). IEEE

[11] Liu, X., Ying, P., Zhong, X., Xu, J., Han, Y., Yu, S., Cai, X.: Highly efficient thermo-optic tunable micro-ring resonator based on an LNOI platform. Optics letters **45**(22), 6318–6321 (2020)

[12] Dehkordi, M.A., Dousti, M., Mirsanei, S.M., Zohoori, S.: A dynamic power-efficient 4 GS/s CMOS comparator. AEU-International Journal of Electronics and Communications **170**, 154812 (2023)

[13] Jaklin, M., García-Lesta, D., Lopez, P., Brea, V.M.: Global shutter CMOS vision sensors and event cameras for on-chip dynamic information. International Journal of Circuit Theory and Applications (2024)

[14] Feldmann, J., Youngblood, N., Karpov, M., Gehring, H., Li, X., Stappers, M., Le Gallo, M., Fu, X., Lukashchuk, A., Raja, A.S., *et al.*: Parallel convolutional processing using an integrated photonic tensor core. Nature **589**(7840), 52–58 (2021)

[15] Ashtiani, F., Geers, A.J., Aflatouni, F.: An on-chip photonic deep neural network for image classification. Nature **606**(7914), 501–506 (2022)

[16] Shen, Y., Harris, N.C., Skirlo, S., Prabhu, M., Baehr-Jones, T., Hochberg, M., Sun, X., Zhao, S., Larochelle, H., Englund, D., *et al.*: Deep learning with coherent nanophotonic circuits. Nature photonics **11**(7), 441–446 (2017)

[17] Zhou, T., Lin, X., Wu, J., Chen, Y., Xie, H., Li, Y., Fan, J., Wu, H., Fang, L., Dai, Q.: Large-scale neuromorphic optoelectronic computing with a reconfigurable diffractive processing unit. Nature Photonics **15**(5), 367–373 (2021)

[18] Xu, X., Tan, M., Corcoran, B., Wu, J., Boes, A., Nguyen, T.G., Chu, S.T., Little, B.E., Hicks, D.G., Morandotti, R., *et al.*: 11 TOPS photonic convolutional accelerator for optical neural networks. Nature **589**(7840), 44–51 (2021)

[19] NVIDIA, N.: H100 tensor core GPU architecture overview (2022)
